# The Mode of Grass Supply to Dairy Cows Impacts on Fatty Acid and Antioxidant Profile of Milk

**DOI:** 10.3390/foods9091256

**Published:** 2020-09-08

**Authors:** Senén De La Torre-Santos, Luis J. Royo, Adela Martínez-Fernández, Cristina Chocarro, Fernando Vicente

**Affiliations:** 1 Servicio Regional de Investigación y Desarrollo Agroalimentario (SERIDA), Carretera AS-267, PK. 19, Villaviciosa, 33300 Asturias, Spain; senendelatorre@gmail.com (S.D.L.T.-S.); ljroyo@serida.org (L.J.R.); admartinez@serida.org (A.M.-F.); 2 ETSEA, Universitat de Lleida, C/Rovira Roure 177, 25198 Lleida, Spain; cristina.chocarro@udl.cat

**Keywords:** grass silage, zero-grazing, grazing, milk, antioxidants, fatty acids

## Abstract

The optimization of milk production includes a rational use of forages, respect for the environment and offers the best quality to consumers. Milk production based on grass and forages produces healthier milk and it is widely spread throughout the Atlantic arc to maximize milk yield per hectare. However, the mode of offering the grass can have a major influence on milk composition. The aim of this study was to evaluate the effect of grass supply mode (grazing, zero-grazing or ensiling) on dairy cows’ performance, with particular reference to fatty acids and fat-soluble antioxidants concentration. A three by three Latin square experiment was performed with 18 dairy cows. Experimental treatments consisted of exclusive feeding with grass silage and zero-grazing, both offered ad libitum indoors, or grazing for 24 h. The results showed that grazing cows had a higher dry matter intake and greater milk yield than cows feeding on grass silage and zero-grazing, as well as higher concentrations of protein, lactose, nonfat-solids and urea in milk than housed cows. Milk fat from grazing cows had a higher proportion of unsaturated fatty acids than from cows feeding on grass silage and zero-grazing, with significant differences in the proportion of vaccenic and rumenic acids. The 18:1 trans-11 to 18:1 trans-10 ratio is proposed as biomarker to identify the milk produced from the management system of grazing cattle. Milk from grazing cows had a greater proportion of lutein than cows eating grass silage, with the zero-grazing system having intermediate values. In conclusion, the mode of grass supply affects fatty acid and antioxidant profiles of milk.

## 1. Introduction

Milk has great importance for humans because of its nutritional characteristics, providing a high nutrient content in relation to its caloric content. The composition of milk determines its nutritional and industrial quality, which affects directly the profitability and competitiveness of milk production [1]. Nowadays, not only the nutritional value, but also other specific components of milk (e.g., fatty acids and vitamins) have attracted interest because of their important relevance in an overall aim to improve the long-term health of consumers and in the added value of milk and its products. Milk composition is the reflection of multiple factors that may or may not be modified through different livestock management practices. The concern of consumers about livestock foods and their methods of production related to the concept of food quality now include, in addition to nutritional value, flavor, aroma and color, as well as ethical aspects such as animal welfare and environment impact of the production system [2]. Consumers assume that a pasture-based dairy cattle feeding is more natural and better aligned with animal welfare requirements than more intensive systems. Knowing the production system can determine the consumers choice at the time of purchase [3]. Thus, Monahan et al. [4] indicated the need to investigate biomarkers that allow associate milk composition with livestock food and, therefore, allow traceability from farm to fork according to production system and geographical origin.

Factors related to nutrition, feeding and management of dairy herds can produce changes in the short term [5]. Many studies show that milk produced from grazing systems has different characteristics in its components than milk produced from confinement systems [6,7]. Fresh forages are important natural source of antioxidants, vitamins and fatty acids in ruminant diets, and their concentrations in forage have an important relationship to the resultant composition and quality of milk and dairy products [8]. Liposoluble antioxidants and fatty acids in milk have been proposed as biomarkers for the authentication of milk produced from pastures, because they depend on external factors that differentiate the production system, diet, exercise and animal welfare [9].

The nutritional quality of milk fat is largely based on its fatty acid profile, which plays a key role in many vital functions and has a direct impact on the health of consumers [10]. In general, the fatty acid content of milk changes in quantity and quality depending on factors related mainly to diet and, to a lesser extent, to the animal and the environment [11,12,13], so that fat content and the fatty acid profile can be indicators of the diet of animals [14] and the management system [6].

The positive impact of liposoluble antioxidants (carotenoids and tocopherols) on human health has been extensively reviewed [15,16,17,18]. The concentration of these bioactive compounds in milk is directly related to dietary concentrations [19]. Fresh pasture is a good source of vitamins and antioxidants that are transferred to milk [20]. Higher levels of antioxidants (α-tocopherol, β-carotene and retinol) have been reported in milk from cows that consume fresh grass compared with diets rich in concentrate or silage [21,22].

The conditions of the oceanic climate in the Atlantic arc favor the production of grass and fodder to feed dairy cattle. Fresh or preserved forages are essential parts of the dairy cows’ diets. A way to help the current profitability problem of milk producers could be guided by the feeding systems that enhance the utilization of their own forage resources. The models based on grazing allow cost savings concerning feeding on the farms [23,24]. Hanson et al. [25] found that dairy farms in the mid-Atlantic region of the USA based on extensive grazing were more profitable than housed dairy farms. However, not all animals have the possibility of grazing, either due to lack of available surface or due to handling difficulties. Thus many farmers have to adopt a cut-and-carry system or provide preserved grass to the manger [26,27].

The objective of the present study was to examine the effectiveness of the delivery method of herbage to dairy cows: grazing, cut-and-carry system (zero-grazing) or grass silage, on milk performance and milk antioxidant and fatty acids profiles with the view to identify biomarkers of the feeding system.

## 2. Materials and Methods

The work was undertaken at the Servicio Regional de Investigación y Desarrollo Agroalimentario (SERIDA) experimental farm (43°28′20″ N, 5°26′10″ W; 10 m above sea level) and adhered to the standard of the EU Animal Welfare Directive Number 2010/63/EU.

### 2.1. Experimental Design and Treatments

Eighteen Holstein cows in the second half of lactation were randomly distributed into three groups of six cows. All cows were selected with an initial average weight of 624 ± 69 kg (average ± standard error), 2.5 ± 1.5 lactations, 108 ± 53 days of lactation and an average daily production of 30.3 ± 7.1 kg of milk. Three treatments were tested: (1) ad libitum grass silage with housed cows (2) ad libitum zero-grazing indoors, and (3) full-time grazing. All treatments were supplemented daily with 6 kg of concentrate offered during milking to meet the energy requirements. Three trials were carried out by following a three by three Latin square design. Each trial lasted 19 days, including 13 days of adaptation to the experimental treatment and six days of sampling and measurements. In each trial, six animals received randomly ad libitum grass silage indoors plus 6 kg of concentrate; ad libitum fresh grass as zero-grazing plus 6 kg of concentrate; or grazing 24 h per day plus 6 kg of concentrate per animal per day with 18 replications per treatment. All forages offered indoor were supplied once a day, and concentrate was offered twice a day.

### 2.2. Experimental Procedure

Grazing and a cut-and-curry system were carried out in three plots with rotational management. The plot size was 1.5 ha with a wide range of grasses: *Poa trivialis* L. (23.15%), *Lolium perenne* L. (16.38%), *Holcus lanatus* L. (14.87%), *Dactylis glomerata* L. (14.26%), *Festuca arundinacea* Scherb. (2.48%), *Agrostis capillaries* L. (1.68%) and *Trisetum flavescens* P. Beauv. (1.21%); legumes: *Trifolium repens* L. (11.80%); species of the family Ranunculaceae: *Ranunculus bulbosus* L. (2.26%), *Ranunculus acris L.* (1.34%); species of the family Asteraceae: *Taraxacum officinale* Weber (1.56%), *Carlina* sp. (1.11%) and other species, all < 1.00% such as *Bromus hordeaceus* L., *Cerastium fontanum* Baumg., *Geranium mole* L., *Poa pratensis* L., *Galium verum* L., *Phleum pratense* L., *Bellis perennis* L., *Carex* sp., *Potentilla erecta* Raeusch., *Tragopogon pratensis* L., *Veronica chamaedrys* L., *Cerastium glomeratum* Thuill., *Lactuca* sp., *Dianthus monspessulanus* L. and *Sonchus soleraceus* L. that together reached 3.09%. Dead matter accounted for the remaining 4.81%. No grass was in bloom. The grass silage offered was from the herbage harvested during the previous spring in the same meadows.

During the experimental period, plots for grazing and zero-grazing were assigned to the corresponding group of animals taking into account the available pregrazing herbage mass. Herbage in the grazing treatment was sampled on the first day of the sampling period by tracing a diagonal transect across the paddock. The sample was composed of five squares (0.20 m^2^ each), leaving a stubble of about 5 cm. Herbage of zero-grazing and grass silage treatments were sampled daily during the experimental period and pooled in one sample by period. Samples of concentrate were taken at the beginning of each experimental period.

### 2.3. Sampling and Chemical Analyses

The individual intake of grass in zero-grazing and silage treatments was automatically recorded daily by an electronic weighing system integrated with a scale pen by a computerized system. Herbage intakes on pasture were estimated using the animal performance method [28]. Briefly, energy requirements were recorded as net energy requirements for maintenance, lactation, body weight changes, walking and grazing. The net energy from herbage intake was estimated as total energy requirements minus the net energy supplied by concentrate intake. Concentrate intake was recorded daily by an automatic feeder coupled to the milking system.

All cows were milked twice daily (at 7:00 and 19:00 h). Milk production was measured and sampled daily in both milking sessions. The two samples from each cow were mixed proportionally according to the milk produced in both milkings.

Forage samples were dried at 60 °C for 24 h to determine the dry matter content, and ground at 0.75 mm. Concentrate samples were milled at 1.00 mm. Feed samples were analyzed for organic matter (OM), crude protein (CP), and neutral detergent fiber (NDF) by near infrared spectroscopy (FOSS NIRSystem 5000, Silver Springs, MD, USA). The energy content was estimated in all samples according to National Research Council (NRC) [29]. The fatty acid and antioxidants concentration of feedstuffs were analyzed in the Dairy Interprofessional Laboratory of Galicia (LIGAL). Extraction and methylation of the fatty acids (FA) were carried out simultaneously according to Sukhija and Palmquist methodology [30]. Esterification of FA was performed using a toluene and methanolic hydrochloric acid solution as follows: heating at 70 °C in a water bath for 2 h, cooling at room temperature and adding 2 mL of hexane and 5 mL of K_2_CO_3_ (6% *w*/*v*) and centrifuging for 5 min at 2500 rpm. The organic phase was immediately evaporated in a nitrogen stream to obtain an oily residue and dissolved in 0.8 mL of hexane. FA methyl esters were separated, identified and quantified using a TRACE GC Ultra equipment (Thermo Fisher Scientific, Waltham, MA, USA) with flame ionization detector (FID), using a 100 m × 0.25 mm i.d. fused silica capillary column (SP-2560 Capillary GC Column, Sigma-Aldrich Inc., Saint Louis, MO, USA). Helium was used as carrier gas at a flow rate of 0.6 mL/min. The temperature of the injector and detector were 250 and 260 °C, respectively. The injection volume was 1 µL. The initial column temperature was set at 140 °C for 5 min; from 140 to 200 °C at 3 °C/min and held for 5 min; from 200 to 240 °C at 3 °C/min and held for 5 min and, finally, held for 38 min. Individual FA were quantified through internal calibration using methylated 9:0, 17:1, 19:0 and 20:2 fatty acids as internal standards.

The samples for antioxidant assay were immediately vacuum packed and frozen (−20 °C) and analyzed according to Chauveau-Duriot et al. [31]. Samples were treated with liquid nitrogen in a Robot Coupe R6 grinder (Vincennes, France). The processing of samples was performed in dim light, using opaque glass. Butylhydroxytoluene (0.1% *v*/*v*) was added as antioxidant, NaHCO_3_ as neutralizing agent, and 10 ppm of echinenone and 3 ppm of δ-tocopherol as internal standards. The lipophilic components were extracted by washing three times with acetone. The analytes were extracted with petroleum ether, the organic phase was evaporated under a nitrogen stream and the dry residue was saponified with KOH in MeOH (5.5% *w*/*v*) for 15 min at room temperature. After centrifugation at 1000× *g* for 5 min at room temperature, the organic phase was collected, evaporated again and reconstituted in the mobile phase. Finally, it was filtered through a syringe filter (Acrodisc Syringe Filter GHP, 25 mm, 0.2 µm, Waters, MA, USA) and transferred into a high performance liquid chromatography (HPLC) vial. An HPLC (Alliance 2695, Waters, MA, USA) system equipped with two serial detectors, UV-Vis and fluorescence, was used for the simultaneous detection and separation of xanthophylls, carotenes and vitamins A and E. Separation of antioxidant was carried out using a reverse phase column RP C18 Kinetex 2.6 µm 4.6 × 150 mm (Phenomenex, Torrance, CA, USA). The sample and column were kept refrigerated at 10 and 13 °C, respectively. The elution of components in the column was performed using a flow of 0.6 mL/min and a quaternary gradient of mobile phase. Quantification was carried out using external calibration models, quantifying the fat-soluble antioxidants according to the recovery factor of both internal standards.

Milk samples were analyzed for fat, protein, lactose, nonfat solids and urea by MilkoScan FT6000 (FOSS Tecctor, Millerd, Denmark) in the Dairy Interprofessional Laboratory of Asturias (LILA). The milk samples for fatty acid and antioxidants analysis were immediately frozen (−20 °C) until their analysis in the Dairy Interprofessional Laboratory of Galicia (LIGAL). Fatty acids were analyzed according to standard methods ISO14156:2001/IDF172 for lipids extraction and ISO15884:2002/IDF182 for preparation of fatty acid methyl esters. Twenty mL of milk were mixed with 96% ethanol, 30% ammonia solution and diethyl ether. After shaking for 1 min, the mixture was left to stand to achieve phase separation then hexane was added, mixed carefully, and left to stand for a second phase separation. Finally, the aqueous layer was discarded. Sodium sulfate solution (10% *w*/*v*) was added to the remaining content, mixed carefully again, left to stand for a third phase separation and, thereafter, the aqueous layer was discarded. The organic layer was transferred to a conical flask, mixed with anhydrous sodium sulfate, left to stand for 10 min and filtered. Finally, the filtrate was evaporated in a rotary steamer (Buchi R-114, Flawil, Switzerland) under a nitrogen stream in a water bath set at 50 °C. The extract was dissolved in hexane, saponified as described above and 0.5 g of sodium hydrogen sulfate were added. Finally, it was centrifuged at 1000× *g* for 5 min at room temperature. FA methyl esters were separated, identified and quantified using a Varian 3900 GC (Varian Inc., Palo Alto, CA, USA) with a flame ionization detector (FID), using a 120 m × 0.25 mm i.d. capillary column (BPX70 GC column, Thermo Fisher Scientific, Waltham, MA, USA). Helium was used as carrier gas at a flow rate of 1.3 mL/min. The temperature of the injector and detector were 250 °C. The initial column temperature was 45 °C for 5 min; from 45 to 175 °C at 13 °C/min and held for 27 min; from 175 to 215 °C at 4 °C/min and held for 35 min. Individual FA peaks were identified by comparison of their retention times with those of pure methyl ester standards (Supelco 37 Component FAME Mix and TVA methyl standard of Supelco Inc., Saint Louis, MO, USA, and methyl CLAc9t11 of Matreya LCC., State College, PA, USA). Individual FA were quantified using an internal calibration using methylated 9:0, 17:1c10, 18:2c12t10 (Matreya LCC., State College, PA, USA) and 19:0 (Sigma-Aldrich, Saint Louis, MO, USA).

Carotenoids and vitamins E and A present in the milk were determined according to the methodology proposed by Gentili et al. [32]. Milk samples were thawed the day before analysis and tempered previous to simultaneous extraction of carotenoids and vitamins. Sample preparation and identification and quantification of antioxidants were carried out according to the methodology described in foods antioxidants analysis.

### 2.4. Statistical Analysis

Statistical analysis was performed using the R statistical package [33]. Dry matter intake, milk yield and milk composition data were analyzed by a general linear model (GLM) procedure using repeated measures ANOVA by following a 3 × 3 Latin square design. The mode of supply of herbage (grazing, zero-grazing or silage grass) was considered as the fixed effect, and period and animal were considered as random effects. Individual animals were considered as experimental units. Significance was set at *p* < 0.05. When the ANOVA was significant, means were separated by Tukey’s test pairwise comparison.

## 3. Results

Table 1 shows the nutritive composition of fresh forage used in grazing and zero-grazing treatments, and grass silage and concentrate used in all treatments. It was not possible to complete the fatty acid and antioxidant profiles analysis of grass used in zero-grazing treatment due to a problem with the samples’ conservation, so these results were not included. The average of crude protein in fresh forage was higher than grass in zero-grazing and grass silage. The energy content of grass silage was lower than grass used in grazing and zero-grazing treatments. In general, the fatty acid and antioxidant concentrations were higher in fresh herbage than grass silage.

The results of dry matter intake and milk production and composition are presented in Table 2. The dry matter intake of forage was higher (*p* < 0.001) in the grazing system than in both housed systems. There were no differences in dry matter intake of concentrate among treatments. The yield was different among systems (*p* < 0.001). The greatest milk production was observed in the grazing feeding system, with intermediate values in the zero-grazing, and the lowest in the grass silage treatment. Grazing milk had the highest proportion of protein, lactose, nonfat solids and urea (*p* < 0.001) compared to both treatments with housed cows. There were no differences in fat proportion among feeding systems.

The milk fatty acid profile is shown in Table 3. Grazing cows presented a higher proportion of vaccenic acid (18:1 trans-11, *p* < 0.05) and rumenic acid (18:2 cis-9 trans-11, *p* < 0.01), as well as a higher (*p* < 0.05) 18:1 trans-11 to 18:1 trans-10 ratio than zero-grazing and grass silage treatments. The milk of the silage system had a higher (*p* < 0.05) proportion of linoleic acid (18:2 cis-9 cis-12) than the grazing system, while there were no differences among treatment in α-linolenic acid (18:3 cis-9 cis-12 cis-15) proportions. There were no differences in the proportion of monounsaturated, saturated and polyunsaturated fatty acids, between unsaturated and saturated fatty acids or n-6 to n-3 ratios among treatments.

The content of fat-soluble antioxidants according to the mode of herbage supply to cows is shown in Table 4. No effect of feeding method was observed in vitamins A and E in milk. Milk from grazing cows had a greater proportion of lutein than milk from cows offered grass silage (*p* < 0.01), with the zero-grazing system showing intermediate values. There were no differences among treatment in other carotenoids and carotenes.

## 4. Discussion

The model of milk production has changed in the western world in the last years and involves intensification of production with an increase in inputs that is not reflected in an increase in the price and quality of milk [34]. Although prices and availability of feed ingredients vary by regions, in the Atlantic arc the production of pastures and legumes used to feed dairy cattle is a great way to decrease the feed diets’ expenses [35].

Energy intake is often a primary limitation for milk yield in grazing cows, even for pastures with high-quality energy content [36]. A good diet formulation strategy should be determined according to ingredient availability. In this study, herbage was used as the only forage source in order to avoid other variation factors. Forage is rarely offered as the sole feed to lactating dairy cows because the dry matter intake (DMI) is generally too low to meet the requirements. The theoretical DMI capacity of dairy cows is approximately 21 kg DM/day [29]. According to our own previous experience, around 15 kg DM/day is the voluntary intake of forage for this type of animal. Consequently, six kilograms (fresh matter basis) of concentrate were offered daily in order to meet the energy requirements for dairy cows with these features (approximately 210 MJ metabolizable energy per day [29]) and a forage to concentrate ratio of 75:25. Nevertheless, only the cows in the grazing treatment reached a forage intake up to 14 kg DM/day, while indoor treatments barely reached 11 kg DM/day. The lower DMI of grass silage treatment than that of the grazing treatment has been previously reported [37], because voluntary intake of silage is lower than that of the herbage from which it was produced. The higher DMI in grazing cows compared with zero-grazing cows could be associated with a greater chance of grazing cows to select better botanical species and the best parts of plants, while housed zero-grazing cows do not have that opportunity. Grazing cows can more readily reject unsuitable forage and preferentially consume more easily digestible portions of grass. Furthermore, the dairy cows consumed just the half of the concentrate offered in all of the treatments tested. The concentrate was offered to the animals twice daily (7:00 and 19:00 h) at the time of milking. This fact could have induced a ruminal subacute acidosis that could lead to a drop in the intake of forage subsequently offered, in spite of maintaining the forage to concentrate ratio. With these levels of intake, is not possible to meet the energetic requirements for cows producing 30 kg milk/day. The energy deficit has been quantified as 25, 53 and 75 MJ/d for grazing, zero-grazing and grass silage treatments, respectively. For this reason, there was a decrease in milk production observed with respect to the values before the beginning of the assay.

The grass supply modes studied in this experiment exhibited a great variation in milk production, milk composition and fatty acid and antioxidant profiles, particularly in lutein content, which are strongly related to the specific compounds from feeds. Grazing cows had a higher DMI than housed cows offered zero-grazing or grass silage. These differences had a significant effect on milk production and milk composition, with a higher concentration of protein, lactose and nonfat solids in the milk of the grazing cows than in the housed cows. In contrast, other studies have shown lower DMI at pasture [38,39] and a consequent drop in milk yield in grazing cows compared to housed cows [40]. However, it should be taken into account that the housed cows in those studies were feeding with balanced total mixed rations while in our study grass or grass silage was the only ingredient of the ration. Furthermore, in our study conditions, grazing cows had the possibility to readily reject unsuitable forage and preferentially consume the leafy and more easily digestible portions of the grass with, possibly, better nutritional value [37]. Other authors showed that it could be possible to maximize energy intake including forages with high NDF digestibility which minimize filling effects and increase milk yield [35,41]. The protein concentration of milk in grazing cows was unchanged with respect to the initial situation, presumably because energy intake was similar to energy requirements, and may be due to a higher duodenal flow of microbial protein and total amino acids [37]. However, grazing cows have a greater concentration of urea in milk than in indoor treatments, in all of the cases in the normal range. This can be explained because the high concentration of protein in meadow grass could have increased the rate of microbial protein synthesis, as well as the concentration of propionate in the rumen, resulting in an increase in milk protein [42]. This can be explained because grazing animals would first select green shoots and the best parts of the plant, which can have great rumen degradability of protein [36], so an excess of urea could accumulate in the rumen that would be absorbed and excreted.

It is well-known that the concentration of lactose is rarely influenced by feeding. However, the results showed a higher lactose concentration in milk from grazing cows. A possible explanation might be related to the increased forage proportion, of up to 80%, which may lead to proliferation of cellulolytic rumen bacteria leading to more propionic acid and, eventually, an increase in the concentration of milk lactose content [43]. Increasing propionate is essential for promoting energy availability for milk production and increasing glucose and lactose synthesis [44]. In addition, as fresh herbage contains a high concentration of sugar, the synthesis of lactic acid in the rumen is favored, which in turn results in a high lactose content in milk. According to the study of Argamentería et al. [45], an increase in the energy supplied in the diet can be associated with an increase in the proportion of lactose.

Some authors have observed that milk produced by grazing cows has a higher fat content than milk produced under a semi-extensive or intensive system [46,47], although other studies have shown opposite results [5,36]. Among hundreds of fatty acids present in bovine milk fat, just a limited number is important because of their relation to nutritional, sensorial and technological properties [48]. The proportion of some transunsaturated fatty acids in bovine milk fat ranges from 2% to 8% of total fatty acids. Among them, the most interest has been focused mainly on transvaccenic acid and conjugated linoleic acid (CLA), and especially rumenic acid because it is the most abundant CLA isomer, for their important antisclerotic, antiatherogenic and anticarcinogenic properties [48,49].

Variations among treatments in the milk fatty acid profile were found. A higher proportion of transvaccenic and rumenic acids, as well as a higher 18:1 trans-11 to 18:1 trans-10 ratio, could be explained in grazing treatments by the proportion of fresh forage in the diet, which is associated with a higher intake of polyunsaturated fatty acids (PUFA) from fresh grass [50]. In addition, an increase in the losses of unsaturated fatty acids (UFA) and total fatty acids (FA) is produced during the wilting and silage processes [48]. Furthermore, the biohydrogenation of 18:2 n-6 could be affected by the high PUFA content in the rumen [51] and, as a consequence, could increase with higher contents of these fatty acids [14]. These results show that the fatty acid profile varies depending on the production system, with a greater proportion of UFA in cows fed on pasture [5]. A direct relationship in the proportion of UFA and decrease in the proportion of saturated fatty acids (SFA) is associated with increasing the proportion of fresh pasture in the cow diet [20]. Differentiation between milk from cows fed fresh grass indoors (zero-grazing) and grazing outdoors has proven difficult. Milk FA profile is different and may be healthier in grazing cows than when cows are stall-fed [52]. Lesser proportions of CLA and 18:1 trans FA in milk from zero-grazing compared to grazing treatments could be explained by the lower DMI. In addition, PUFA losses have been reported in grass immediately after cutting in fresh and preserved forages by oxidative processes of plant tissues [48]. Therefore, fatty acid intake is affected. Other factors such as intake pattern, or possibilities for feed selection by the cow, could also play a role. In our study higher trans isomers of monounsaturated fatty acids (MUFA) proportion and higher 18:1 trans-11 to 18:1 trans-10 ratio have been observed in milk from grazing cows compared to zero-grazing treatments. The 18:1 trans-11 to 18:1 trans-10 ratio increases when fresh forage proportion increases in the diet [53]. Consequently, the 18:1 trans-11 to 18:1 trans-10 ratio can be proposed to identify grass-fed milk.

Fat-soluble antioxidants and vitamins present in cows’ milk are derived specially from green forage [54,55]. Milk and dairy products are a rich source of carotenoids and bioavailable vitamin A in the daily diet of consumers [56]. However, they have low levels of vitamin E. Vitamin E plays an important role as an antioxidant that protects milk fat against autoxidation [57]. Beneficial effects related to the reduction of oxidative stress, which was shown to be a risk factor for a wide range of chronic disease processes including cardiovascular disease, cancer, neurodegenerative diseases, impaired immunity and premature ageing, are associated with consumption of diets with high antioxidant contents [17,56]. 

The variability in milk content of carotenoids and vitamins has been associated with the presence of grass in the ration and its levels in the grass are highly related to drying and preservation because of light exposure [22]. Different studies reported that the content of β-carotene and fat-soluble vitamins were up to four times higher in the milk of grazing cows compared to the milk of cows offered total mixed rations or a high proportion of concentrate [56,58]. Milk produced from pasture is yellower in color as a result of the higher β-carotene concentration of the milk [58]. Our results did not show wide differences in the antioxidant profile among treatments. Lutein was the only antioxidant in milk that showed significant differences. It could be because all treatments were based on meadows with very similar botanical species and a low biodiversity.

## 5. Conclusions

In conclusion, the modification of milk composition is associated with the feeding system. It is possible to distinguish from cows in a grazing feeding system by significant variability in fatty acids profile, as well as lutein content. Both were associated with the presence of fresh grass in the diet, especially when grass is consumed as grazing. Similarly, under the conditions of the experiment, grazing dairy cows had higher milk production and higher concentrations of protein, lactose, nonfat solids, urea and lutein, as well as unsaturated fatty acids. The 18:1 trans-11:18 to 1 trans-10 ratio is proposed as biomarker to identify milk produce from the management system of grazing cattle.

## Figures and Tables

**Table 1:** Nutritive value, metabolizable energy, fatty acid and antioxidants profiles of forage used in grazing, zero-grazing, grass silage treatments and concentrate used in all treatments.

Item	Grazing	Zero-Grazing	Grass Silage	Concentrate
Dry matter (DM, %)	21.18	24.04	30.52	87.71
Organic matter (% DM)	87.45	91.15	89.47	91.43
Crude protein (% DM)	15.12	10.86	9.86	20.33
Neutral detergent fiber (% DM)	57.40	58.97	67.87	19.31
Metabolizable energy (MJ/kg DM)	9.56	9.29	8.27	12.74
Fatty Acids (g/100 g fatty acids)				
10:1 cis-9	0.28	NA ^1^	0.06	0.02
11:0	0.21	NA	0.84	0.01
12:0	0.70	NA	1.12	0.18
13:0	0.86	NA	0.88	0.01
14:0	0.62	NA	0.80	0.49
15:0	0.14	NA	0.33	0.05
15:1 cis-10	1.33	NA	1.14	0.00
16:0	18.19	NA	19.97	25.33
16:1 cis-7 + 16:1 trans-9	2.31	NA	1.43	0.05
16:1 cis-9	0.27	NA	1.16	0.14
17:0	0.28	NA	0.51	0.10
18:0	1.98	NA	2.61	2.77
18:1 cis-9	3.74	NA	8.98	28.09
18:2 cis-9 cis-12	16.50	NA	28.22	39.15
18:3 cis-6 cis-9 cis-12	0.11	NA	0.19	0.05
18:3 cis-9 cis-12 cis-15	49.05	NA	26.76	2.37
20:0	0.73	NA	0.87	0.29
20:1 cis-9	0.24	NA	0.32	0.25
20:1 cis-11	0.31	NA	0.50	0.35
21:0	0.17	NA	0.32	0.05
22:0	1.08	NA	1.49	0.13
23:0	0.15	NA	0.35	0.03
24:0	0.65	NA	1.05	0.09
24:1	0.11	NA	0.10	0.01
∑ SFA ^2^	25.75	NA	31.14	29.52
∑ MUFA ^3^	8.58	NA	13.69	28.91
∑ PUFA ^4^	65.67	NA	55.17	41.56
PUFA to SFA ratio	2.55	NA	1.77	1.41
n-6 to n-3 ratio	0.34	NA	1.05	16.54
Antioxidants (mg/kg DM)				
Neoxanthin	14.97	NA	0.44	0.04
Violaxanthin	13.28	NA	0.29	<LQ ^5^
Antheraxanthin	1.58	NA	0.95	0.01
Lutein	62.45	NA	23.79	0.43
Zeaxanthin	3.21	NA	1.49	0.07
Β-Cryptoxanthin	0.57	NA	0.18	0.04
∑-trans-β-Carotenes	30.81	NA	7.17	0.09
9-cis-β-Carotenes	6.28	NA	2.13	0.06
13-cis-β-Carotenes	3.44	NA	0.82	0.08
α-tocopherol	9.64	NA	8.45	2.83
γ-tocopherol	1.60	NA	1.03	4.29

^1^ not analyzed. ^2^ Saturated fatty acids. ^3^ Monounsaturated fatty acids. ^4^ Polyunsaturated fatty acids. ^5^ <LQ, below quantification level.

**Table 2:** Food intake, milk yield and milk composition according to mode of supply of herbage to dairy cows: grazing, zero-grazing or grass silage.

Item	Grazing	Zero-Grazing	Grass Silage	RSD	*p* ^2^
Forage (kg DM/d)	14.34 ^a^	11.54 ^b^	10.54 ^b^	2.40	***
Concentrate (kg DM/d)	3.75	3.66	3.56	0.62	NS
Milk (kg/d)	23.4 ^a^	18.1 ^b^	14.0 ^c^	3,57	***
Fat (g/kg)	35.8	33.7	36.1	3.18	NS
Protein (g/kg)	32.1 ^a^	29.1 ^b^	27.8 ^b^	2.90	***
Lactose (g/kg)	45.7 ^a^	41.0 ^b^	41.7 ^b^	2.56	***
SNF ^1^ (g/kg)	83.9 ^a^	77.8 ^b^	76.3 ^b^	3.79	***
Urea (mg/kg)	281 ^a^	200 ^b^	215 ^b^	40.3	***

^1^ Solids nonfat. ^2^ Statistical significance: NS: *p* > 0.05, *: *p* < 0.05, **: *p* < 0.01, ***: *p* < 0.001. ^a^, ^b^, ^c^ Means followed by different lowercase letters are significantly different.

**Table 3:** Fatty acids profile in milk according to mode of supply of herbage to dairy cows: grazing, zero-grazing or grass silage.

Item (g/100 g Fatty Acids)	Grazing	Zero-Grazing	Grass Silage	SD ^1^	*p* ^2^
4:0	5.57	5.36	5.31	0.286	NS
6:0	1.84 ^a^	1.89 ^a^	1.71 ^b^	0.053	*
8:0	0.97	0.99	0.85	0.061	NS
10:0	2.07	2.11	1.71	0.223	NS
10:1 cis-9	0.06	0.06	0.05	0.005	NS
11:0	0.03	0.02	0.01	0.009	NS
12:0	2.14	2.45	1.97	0.271	NS
13:0	0.09	0.09	0.08	0.009	NS
14:0	9.82	10.14	8.98	0.699	NS
14:0 iso	0.22	0.20	0.21	0.010	NS
14:1 cis-9	1.00	1.13	0.92	0.104	NS
15:0	1.21	1.24	1.17	0.046	NS
15:0 iso	0.46	0.47	0.42	0.035	NS
15:0 anteiso	0.74 ^a^	0.71 ^a^	0.59 ^b^	0.040	*
15:1 cis-10	0.01	0.01	0.01	0.003	NS
16:0	27.86	30.73	29.94	1.031	NS
16:1 cis-9	1.80	2.17	2.20	0.180	NS
17:0	0.58	0.64	0.65	0.028	NS
18:0	9.89	9.57	8.85	0.746	NS
18:1 trans-6 + 18:1 trans-9	0.50 ^a^	0.41 ^b^	0.39 ^b^	0.024	*
18:1 trans-10	0.27 ^a^	0.21 ^b^	0.20 ^b^	0.025	*
18:1 trans-11	5.08 ^a^	2.77 ^b^	2.01 ^b^	0.745	*
18:1 trans-12	0.20 ^a^	0.15 ^b^	0.14 ^b^	0.015	*
18:1 cis-9	21.59	21.97	25.52	1.521	NS
18:1 cis-11	0.37 ^b^	0.44 ^b^	0.57 ^a^	0.051	*
18:1 cis-12	0.06 ^b^	0.06a ^b^	0.07 ^a^	0.004	*
18:2 tran-9 trans-12	0.07 ^a^	0.07 ^a^	0.05 ^b^	0.003	**
18:2 cis-9, cis-12	1.16 ^b^	1.38 ^ab^	1.60 ^a^	0.124	*
18:2 cis-9 trans-11 (CLA ^3^)	2.29 ^a^	1.37 ^b^	1.05 ^b^	0.218	**
Other isomers CLA	0.21	0.24	0.24	0.015	NS
18:3 cis-9 cis-12 cis-15	0.55	0.47	0.43	0.070	NS
18:3 cis-6 cis-9 cis-12	0.03	0.03	0.03	0.003	NS
20:0	0.21 ^b^	0.25 ^a^	0.27 ^a^	0.012	**
20:3	0.15 ^b^	0.22 ^ab^	0.27 ^a^	0.030	*
20:5	0.01	0.01	0.01	0.006	NS
20:2	0.01 ^c^	0.02 ^b^	0.03 ^a^	0.001	***
20:3	0.08	0.10	0.10	0.011	NS
20:4	0.01	0.02	0.02	0.004	NS
20:1 cis-11	0.04	0.03	0.05	0.006	NS
21:0	0.07	0.08	0.08	0.005	NS
22:0	0.07	0.08	0.07	0.006	NS
22:5	0.10 ^b^	0.11 ^b^	0.15 ^a^	0.012	*
22:6	0.01	0.01	0.01	0.009	NS
22:2	0.08	0.09	0.10	0.013	NS
23:0	0.05	0.06	0.05	0.006	NS
24:0	0.07	0.08	0.07	0.006	NS
Sum of fatty acids					
∑ SFA ^4^	62.81	65.07	62.49	1.479	NS
∑ BCFA ^5^	1.43 ^a^	1.39 ^a^	1.22 ^b^	0.053	*
∑ MUFA ^6^	30.98	29.43	32.16	1.417	NS
∑ cis-MUFA	24.93	25.89	29.41	1.743	NS
∑ trans-MUFA	6.05 ^a^	3.53 ^b^	2.75 ^b^	0.791	*
∑ PUFA ^7^	4.78	4.11	4.13	0.271	NS
∑ n-6	1.45 ^b^	1.70 ^ab^	1.92 ^a^	0.142	*
∑ n-3	0.83	0.79	1.00	0.062	NS
Ratios					
PUFA to SFA	0.08	0.06	0.07	0.007	NS
UFA to SFA	0.57	0.52	0.58	0.037	NS
18:1 trans-11 to 18:1 trans-10	18.34 ^a^	13.08 ^b^	10.35 ^b^	1.912	*
n-6 to n-3	1.77	2.17	2.10	0.189	NS

^1^ Standard deviation. ^2^ Statistical significance: NS: *p* > 0.05, *: *p* < 0.05, **: *p* < 0.01, ***: *p* < 0.001. ^3^ Conjugated linoleic acid. ^4^ Saturated fatty acids. ^5^ Branched chain fatty acids ^6^ Monounsaturated fatty acids. ^7^ Polyunsaturated fatty acids. ^a^, ^b^, ^c^ Means followed by different lowercase letters are significantly different.

**Table 4:** Fat-soluble antioxidants composition according to mode of supply of herbage to dairy cows: grazing, zero-grazing or grass silage.

Item (µg/L Milk)	Grazing	Zero-Grazing	Grass Silage	SD ^1^	*p* ^2^
Retinol	855	852	827	202.6	NS
α-Tocopherol	1189	962	1068	237.5	NS
γ-Tocopherol	17.8	19.9	23.3	3.50	NS
Lutein	21.9 ^a^	15.5 ^b^	9.1^c^	3.19	**
Zeaxanthin	1.19	0.42	0.47	0.332	NS
β-Cryptoxanthin	3.13	1.64	1.55	1.031	NS
All-trans-β-Carotene	255	184	179	45.6	NS
9-cis-β-Carotene	1.73	2.05	1.60	0.775	NS
13-cis-β-Carotene	9.52	7.08	6.82	1.540	NS

^1^ Standard deviation. ^2^ Statistical significance: NS: *p* > 0.05, *: *p* < 0.05, **: *p* < 0.01, ***: *p* < 0.001. ^a^, ^b^, ^c^ Means followed by different lowercase letters are significantly different.
